# Comparative evaluation of ChatGPT and Gemini feedback in scenario-based clinical reasoning among medical interns

**DOI:** 10.1590/1806-9282.20260869

**Published:** 2026-10-12

**Authors:** Hilal Hatice Ülkü, Fulya Torun, Selcen Öncü

**Affiliations:** 1 Aydın Adnan Menderes University, Aydın Vocational School – Aydın, Turkey.; 2 Aydın Adnan Menderes University, Faculty of Education, Department of Computer and Instructional Technologies – Aydın, Turkey.; 3 Aydın Adnan Menderes University, Faculty of Medicine, Department of Medical Education – Aydın, Turkey.

**Keywords:** Artificial intelligence, Clinical reasoning, Feedback, Medical education, Patient simulation

## Abstract

**OBJECTIVE:**

Artificial intelligence tools are increasingly used to provide individualized feedback in medical education. The aim of this study was to compare the quality of feedback generated by ChatGPT and Gemini during scenario-based clinical reasoning activities.

**METHODS:**

A convergent mixed-methods study was conducted with 32 medical interns randomly assigned to ChatGPT (n=16) or Gemini (n=16). Following a virtual patient interview on iron deficiency anemia, artificial intelligence-generated feedback was evaluated by interns and medical education specialists using an 11-item rubric assessing clarity, accuracy, structure, identification of strengths and weaknesses, contribution to clinical reasoning, and overall educational value. Quantitative data were analyzed using descriptive statistics and Mann-Whitney U tests, while qualitative data were examined through content analysis.

**RESULTS:**

Interns rated ChatGPT slightly higher than Gemini (1.80±0.20 vs. 1.75±0.26), whereas specialists reported similar scores (1.91±0.06 vs. 1.87±0.10). No statistically significant differences were identified (p>0.05). Both systems were perceived as clear, supportive, and constructive; however, insufficient patient-centeredness emerged as a common limitation of both artificial intelligence systems. Qualitative findings indicated that ChatGPT provided more structured and reflective feedback, whereas Gemini offered more concrete examples. Common limitations included insufficient patient-centeredness, lack of systematicity, and occasional unrealistic statements.

**CONCLUSION:**

ChatGPT and Gemini generated educationally useful feedback for scenario-based clinical reasoning activities. Although artificial intelligence tools cannot replace expert supervision, they may support personalized learning and reduce faculty workload when used under human oversight.

## BACKGROUND

Clinical reasoning is a core competency in medical education and is particularly important during internship, when students integrate theoretical knowledge with patient care responsibilities. Scenario-based case analysis is widely used to support clinical reasoning and decision-making skills, and effective feedback is essential for maximizing learning outcomes. High-quality feedback helps learners identify strengths and weaknesses, promotes reflection, and supports professional development^1–3^.

Recent advances in artificial intelligence (AI), particularly large language models (LLMs), have created new opportunities for providing individualized educational feedback. AI-based systems can analyze learner responses, generate personalized comments, and offer suggestions for improvement in a timely manner. These features may be especially valuable in medical education, where increasing student numbers and faculty workload often limit opportunities for individualized feedback^4–7^.

Despite these advantages, concerns remain regarding the educational quality of AI-generated feedback. Previous studies have highlighted issues related to contextual understanding, consistency, transparency, and the ability of AI systems to accurately reflect clinical performance. Therefore, AI-generated feedback should be evaluated not only from a technical perspective but also in terms of its educational value and relevance to clinical learning^8–10^.

Among the most widely used AI tools, ChatGPT and Gemini have demonstrated considerable potential in supporting learning activities. However, despite their widespread use, evidence directly comparing the educational quality of feedback generated by these two LLMs during scenario-based clinical reasoning activities remains scarce. To the best of our knowledge, no previous study has directly compared ChatGPT and Gemini in this specific educational context from the perspectives of both medical interns and medical education specialists. Understanding how learners and educators perceive AI-generated feedback is important for determining the role of these technologies in medical education.

Therefore, this study aimed to compare the quality of feedback generated by ChatGPT and Gemini during scenario-based clinical reasoning activities among medical interns. Specifically, feedback quality was evaluated by both medical interns and medical education specialists, and qualitative analyses were conducted to identify strengths and areas for improvement.

## METHODS

### Study design

A convergent mixed-methods study was conducted to evaluate the quality of feedback generated by two AI tools, ChatGPT and Gemini, during scenario-based clinical reasoning activities. Quantitative and qualitative data were collected concurrently and integrated during interpretation.

### Participants

The study included 32 final-year medical interns from Aydın Adnan Menderes University, Türkiye. Participants were randomly assigned to either ChatGPT (n=16) or Gemini (n=16). Because this was an exploratory educational study, a convenience sample consisting of all eligible interns who volunteered during the study period was included. No formal a priori sample size calculation or power analysis was performed. In addition, two medical education specialists independently evaluated all AI-generated feedback.

### Data collection

A structured virtual patient scenario on iron deficiency anemia was developed based on national undergraduate medical education competencies and reviewed by experts in internal medicine and family medicine. A standardized virtual patient scenario, interview prompt, and feedback prompt were developed and pilot-tested before implementation to ensure consistency across AI interactions. The complete scenario and standardized prompts used for both ChatGPT-4o and Gemini 2.5 Flash are provided in Supplementary File 1.

ChatGPT-4o and Gemini 2.5 Flash were used as AI tools. Each participant completed a written virtual patient interview with the assigned AI system. Following the interaction, the AI generated individualized feedback regarding the participant's clinical reasoning and patient interview performance. These AI-generated feedback reports subsequently constituted the evaluation material for both the interns and the medical education specialists.

Feedback quality was assessed using a researcher-developed rubric that was developed based on established feedback quality frameworks in medical education and reviewed by two medical education experts for content validity. The rubric consisted of 11 items evaluating clarity, accuracy, structure, identification of strengths and weaknesses, contribution to clinical reasoning, and overall educational value. Items were scored as "No" (0), "Partly" (1), or "Yes" (2), with higher scores indicating better feedback quality.

The same 11-item rubric was completed by both the medical interns and the medical education specialists. However, their evaluation perspectives differed. Interns evaluated the quality of the AI-generated feedback they personally received following the virtual patient interview, whereas the specialists independently evaluated the educational quality of the AI-generated feedback itself. To minimize potential evaluator bias, all feedback reports were anonymized and presented to the specialists by an independent researcher who was not involved in the evaluation process. Consequently, the specialists remained blinded to whether each feedback report had been generated by ChatGPT-4o or Gemini 2.5 Flash.

For the qualitative component, evaluators provided written comments regarding the overall quality, strengths, and areas for improvement of the feedback.

### Statistical analysis

Descriptive statistics were calculated for all variables. Because data were not normally distributed, comparisons between ChatGPT and Gemini were performed using the Mann-Whitney U test. Statistical significance was set at p<0.05. Analyses were conducted using SPSS software.

Qualitative data were analyzed using content analysis with MAXQDA 2022. Two researchers independently coded the data and discussed discrepancies until consensus was achieved. Percent agreement between coders was 98.3%.

## RESULTS

In total, 32 medical interns participated in the study (ChatGPT=16, Gemini=16). Feedback quality was evaluated by both interns and medical education specialists.

Interns rated ChatGPT slightly higher than Gemini (1.80±0.20 vs. 1.75±0.26), while specialists reported similar scores (1.91±0.06 vs. 1.87±0.10). No statistically significant differences were observed between the two AI tools in either intern or specialist evaluations (all p>0.05) (Table 1). Effect size analysis showed a small difference in intern evaluations (Cohen's d=0.22) and a moderate difference favoring ChatGPT in specialist evaluations (Cohen's d=0.49).

**Table 1:** Interns and medical education specialists’ evaluation of artificial intelligence-generated feedback (mean±standard deviation).

Evaluator	ChatGPT	Gemini	p-value	Cohen's d
Interns	1.80±0.20	1.75±0.26	0.552	0.22
Specialists	1.91±0.06	1.87±0.10	0.410	0.49

Among intern evaluations, the highest-rated characteristics of AI-generated feedback were the use of non-judgmental language, supportive communication, and clear identification of strengths. The lowest-rated rubric criterion for both systems was Descriptiveness, reflecting the tendency of the AI-generated feedback to include inferences beyond information explicitly stated during the interview. Among specialist evaluations, the highest-rated rubric criteria were Clarity and Comprehensibility, Descriptiveness, and Justification and Rationale, whereas Scientific Accuracy and Organization (Systematic Structure) received the lowest ratings for both ChatGPT and Gemini (Table 2).

**Table 2:** Items with the highest and lowest scores.

Evaluator	Highest-rated items	Lowest-rated items
Interns	Supportive language Identification of strengths	Descriptiveness (feedback based only on the student's responses without unsupported inferences)
Specialists	Clarity and comprehensibility; contextual appropriateness (descriptiveness); justification and rationale	Scientific accuracy; organization (systematic structure)

Qualitative analysis identified three major domains: overall feedback quality, strengths, and areas for improvement. Feedback generated by both AI systems was generally perceived as clear, motivating, constructive, and educationally useful. Participants particularly valued the detailed explanations and practical suggestions provided by the systems.

However, several limitations were identified. The most frequently reported concerns included insufficient detail, lack of systematic organization, limited emphasis on communication skills, and inadequate identification of clinical errors. Some feedback was also perceived as unrealistic or insufficiently patient-centered.

Comparison of the two systems revealed distinct characteristics. ChatGPT was perceived as more structured, concise, and reflective, frequently encouraging learners to reconsider their reasoning process. Gemini was viewed as providing more concrete examples and realistic conversational interactions, although patient-centered questioning was less frequently emphasized. A summary of the qualitative findings is presented in Figure 1.

**Figure 1:**
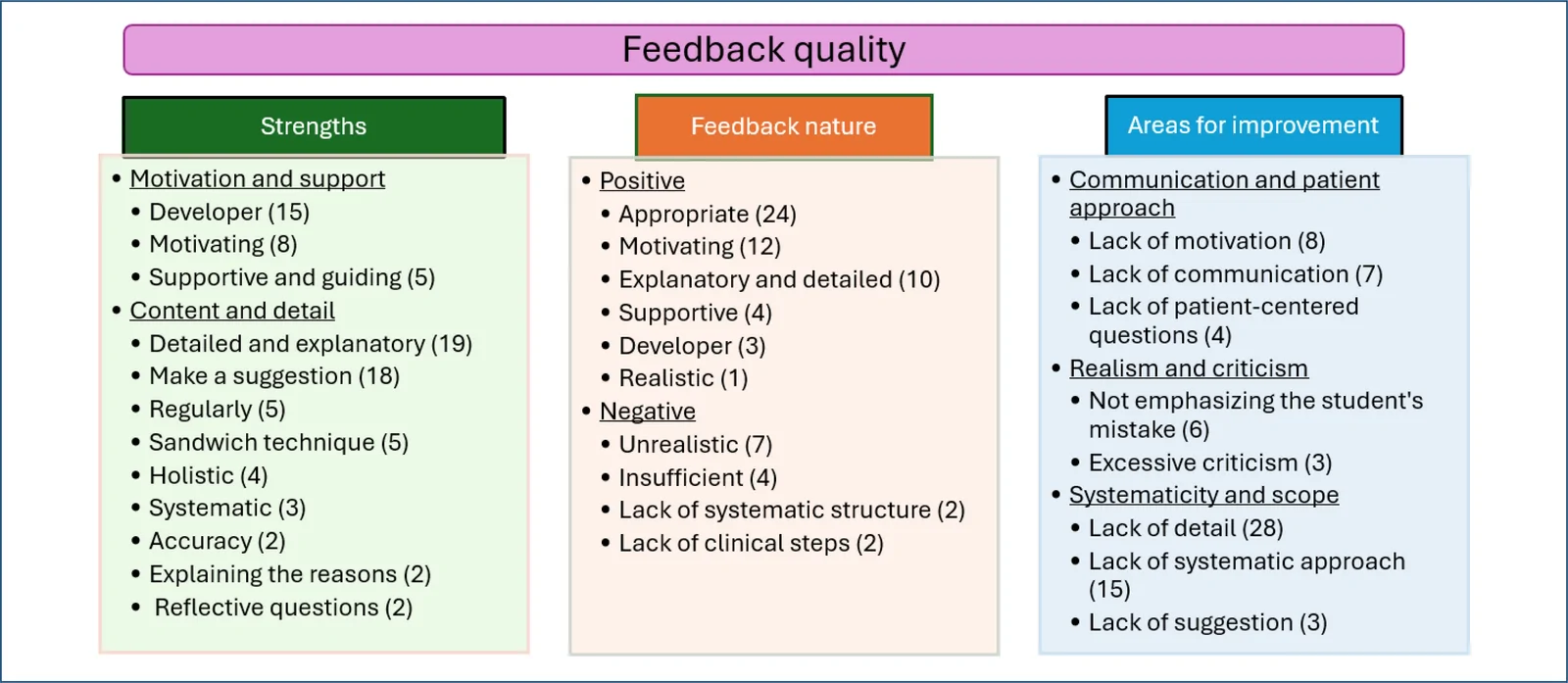
Code distribution of feedback quality.

## DISCUSSION

This study evaluated the quality of feedback generated by ChatGPT and Gemini during scenario-based clinical reasoning activities. Both interns and medical education specialists perceived the feedback as clear, supportive, constructive, and educationally valuable. Although no statistically significant differences were identified between the two AI systems, integrating the quantitative and qualitative findings revealed important differences in the educational characteristics of the feedback generated by each system. While the overall rubric scores were comparable, the qualitative findings provided additional insight into the specific strengths and limitations underlying these quantitative results.

One of the most important findings was that both AI tools were rated highly for clarity, supportive language, and identification of strengths and areas for improvement. Feedback is a critical component of clinical education because it enables learners to recognize performance gaps, reflect on their decisions, and improve future performance. High-quality feedback has been associated with improved self-regulation, motivation, and professional development, whereas poorly structured feedback may reduce learner engagement and limit educational benefit. The positive evaluations obtained from both interns and specialists suggest that AI systems can provide educationally meaningful feedback that aligns with key principles of effective feedback in medical education^3^.

Another notable finding was the perceived personalization of AI-generated feedback. Participants reported that feedback was generally relevant to their performance and frequently included suggestions for improvement. This finding is consistent with previous studies demonstrating that AI systems can adapt feedback to learner responses and support individualized learning experiences^11,12^. Personalized feedback is particularly valuable in medical education, where students progress at different rates and require targeted guidance to develop clinical reasoning skills. The ability of AI systems to provide immediate and individualized responses may help address challenges associated with large student cohorts and limited faculty availability.

The qualitative findings further highlighted the educational value of AI-generated feedback. Both systems were frequently described as explanatory, motivating, and developmental. Participants appreciated feedback that not only identified deficiencies but also offered practical recommendations for improvement. Similar findings have been reported in studies showing that ChatGPT-generated feedback may be perceived as more detailed and easier to understand than traditional feedback approaches^12,13^. These characteristics suggest that AI-based systems may complement existing educational practices by promoting reflection and supporting self-directed learning.

Despite these strengths, several limitations of AI-generated feedback were identified. The lowest-rated item among interns concerned the tendency of AI systems to generate interpretations that extended beyond information explicitly provided during the consultation. This finding suggests that AI systems may occasionally produce assumptions or inferences that are not fully supported by the available data. Similar concerns have been reported in previous studies evaluating LLMs in educational settings^14^. From an educational perspective, such inaccuracies may reduce learner trust and potentially reinforce misconceptions if feedback is accepted uncritically. More importantly, feedback based on assumptions rather than the learner's actual performance may lead to inaccurate educational judgments and inappropriate learning recommendations. These findings reinforce the need for human oversight when AI-generated feedback is used in medical education, particularly in contexts involving formative assessment and clinical reasoning. Therefore, AI-generated feedback should be interpreted carefully and verified before being incorporated into educational decision-making or learner feedback.

Participants also identified limitations related to systematic organization, depth of feedback, and patient-centeredness. Effective clinical feedback should address not only diagnostic reasoning but also communication skills, patient-centered care, and professional behavior. In the present study, both systems were perceived as less effective in emphasizing patient-centered questioning and communication-related aspects of clinical encounters. Given the increasing emphasis on patient-centered care in contemporary medical education, this limitation deserves particular attention. Since communication and patient-centered care are core competencies in competency-based medical education, limitations in these areas may reduce the educational usefulness of AI-generated feedback. AI-generated feedback may be more effective when supplemented by educator guidance that explicitly addresses communication and professionalism competencies.

Another important concern was the limited ability of AI systems to identify and emphasize clinical errors. One of the primary purposes of feedback in clinical education is to help learners recognize mistakes before encountering similar situations in real clinical practice. However, qualitative findings indicated that some errors were insufficiently highlighted or inadequately discussed. Similar limitations have been reported in AI-supported problem-based learning environments, where contextual understanding and clinical nuance remain challenging for LLMs^4^. These findings reinforce the need for human oversight when AI-generated feedback is used in medical training.

Although quantitative comparisons revealed no statistically significant differences between ChatGPT and Gemini, qualitative findings suggested distinct educational characteristics. ChatGPT was generally perceived as more structured, reflective, and supportive. In particular, its tendency to encourage reflection may be beneficial for the development of clinical reasoning and self-assessment skills. Gemini, in contrast, was perceived as providing more concrete examples and realistic conversational interactions. However, patient-centered questioning was less frequently emphasized. These findings suggest that different AI systems may possess unique educational strengths and that their suitability may vary according to instructional goals. Although quantitative comparisons revealed no statistically significant differences, effect size estimates suggested a moderate advantage for ChatGPT in specialist evaluations, indicating potentially greater educational value that warrants further investigation. These qualitative findings also help explain the quantitative results. Although overall rubric scores did not differ significantly between the two AI systems, specialists identified systematic organization as one of the lowest-rated aspects of AI-generated feedback. This finding aligns with the qualitative perception that ChatGPT produced more structured and reflective feedback, which may explain the moderate effect size favoring ChatGPT in specialist evaluations. Likewise, Gemini's emphasis on providing concrete examples and more natural conversational interactions may have contributed to its comparable overall rubric scores despite differences in specific educational characteristics.

The educational implications of these findings are substantial. Previous research has shown that AI-supported feedback systems can improve learning efficiency, facilitate personalized instruction, and reduce faculty workload^15–17^. Our findings support this growing body of evidence and indicate that AI-generated feedback can serve as a valuable supplementary resource in clinical education. However, the limitations identified in this study demonstrate that AI systems should not replace expert educators. Rather, they should be integrated within a hybrid educational model in which AI-generated feedback is reviewed, contextualized, and enriched by human experts. Such an approach may maximize the benefits of AI while minimizing risks associated with inaccuracies, lack of contextual understanding, and insufficient patient-centeredness. In practice, this hybrid model could be implemented by using AI-generated feedback as an initial formative review immediately following a student's virtual patient interview or clinical case presentation. The AI system could provide rapid, structured feedback by identifying strengths, areas for improvement, and preliminary recommendations, after which a faculty member would review, verify, and expand the feedback to ensure scientific accuracy, educational relevance, patient-centeredness, and alignment with curricular learning objectives. This collaborative workflow preserves the educational value of timely AI-supported feedback while maintaining the essential role of educators in fostering clinical reasoning, reflective practice, and professional development.

## LIMITATIONS

This study was conducted at a single medical school with a relatively small convenience sample of interns and a limited number of medical education specialists. In addition, no formal a priori sample size calculation or power analysis was performed. Therefore, the findings should be interpreted with appropriate caution and confirmed in larger, adequately powered studies. In addition, AI-generated outputs are highly dependent on prompt design and model version. Because commercial AI systems evolve rapidly, future versions of ChatGPT and Gemini may produce different results. Another limitation is that this study evaluated the perceived educational quality of AI-generated feedback rather than its direct impact on learning outcomes. Although participants generally perceived the feedback as educationally valuable, the study did not determine whether it resulted in measurable improvements in clinical reasoning, knowledge acquisition, or subsequent learner performance. Furthermore, occasional role-assignment errors and unrealistic responses required researcher intervention during implementation. Future studies should include larger multi-center samples and examine the effects of AI-generated feedback on clinical performance, clinical reasoning, and long-term learning outcomes.

## CONCLUSION

Both ChatGPT- and Gemini-generated feedback were perceived as clear, supportive, and educationally useful by medical interns and medical education specialists. No significant differences were identified between the two systems. Nevertheless, qualitative findings suggested that ChatGPT provided more structured and reflective feedback, whereas Gemini offered more concrete examples. Although AI-generated feedback shows promise as a tool for supporting personalized learning and reducing faculty workload, limitations related to contextual accuracy, patient-centeredness, and consistency remain. Therefore, AI-based feedback should be integrated into medical education not as an independent assessment strategy, but as a complementary resource supported by expert oversight.

## Data Availability

The datasets generated and/or analyzed during the current study are available from the corresponding author upon reasonable request.
